# A multifaceted ecological assessment reveals the invasion of the freshwater red macroalga *Montagnia macrospora* (Batrachospermales, Rhodophyta) in Taiwan

**DOI:** 10.1002/ece3.8906

**Published:** 2022-05-06

**Authors:** Silvia Fontana, Lan‐Wei Yeh, Shing Hei Zhan, Shao‐Lun Liu

**Affiliations:** ^1^ 34890 Department of Life Science & Center for Ecology and Environment Tunghai University Taichung Taiwan; ^2^ 8166 Department of Zoology & Biodiversity Research Centre The University of British Columbia Vancouver British Columbia Canada

**Keywords:** alien species, East Asia, ecological competence, South America

## Abstract

Invasive freshwater macroalgae are rarely described. *Montagnia macrospora* is a freshwater red alga introduced from South America to East Asia via the global aquarium trade. The earliest occurrence record of this alga in Taiwan is dated 2005. To determine whether *M*. *macrospora* has become invasive in Taiwan and to understand the traits that facilitated its invasion, we took a multifaceted approach that combines examination of ecological background and population genetic analysis. Our island‐wide survey showed that *M*. *macrospora* is widespread in the field across Taiwan, where the climate greatly differs from that of South America, and can self‐sustain for nearly a decade. Our population genetic analysis revealed a lack of genetic diversity of *M*. *macrospora* in Taiwan, consistent with the hypothesis that the alga expanded through asexual reproduction. Moreover, during our long‐term ecological assessments and field surveys, we observed that *M*. *macrospora* is an ecological generalist that can survive in a wide range of temperature, pH, illumination, and nutrient enrichment. Taken together, our data suggest that *M*. *macrospora* has successfully invaded the freshwater ecosystems of Taiwan, likely due to its ability to disperse asexually and to grow under broad environmental conditions. We hope that our study brings attention to invasive freshwater algae, which have been overlooked in conservation planning and management.

## Graphical abstract

Studies of freshwater red algae that combine several aspects of freshwater macroalgae biology and invasion biology, such as ours, are rare. Our study fills an important knowledge gap that will aid in the conservation planning and management of invasive freshwater red macroalgae that are overlooked or are yet to be discovered.

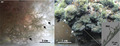

## 1 INTRODUCTION

An alien species is a species introduced from its native range to a non‐native range by human activities. Introduced alien species might cause negative ecological and socioeconomic impacts on the non‐native ranges when they become widespread invasive species (Richardson et al., 2000). Despite the enormous amount of effort and funding spent to limit the spread of invasive species, effective control and eradication of invasive species are seldom successful (reviewed in Simberloff, 2021).

In freshwater environments, many introduced species of fish, vascular plants, and invertebrates have been documented (reviewed in Havel et al., 2015). However, introduced species of freshwater macroalgae have received far less attention, with only a few known invasive cases: the green algae *Hydrodictyon reticulatum* in New Zealand (Hawes et al., 1991) and *Nitellopsis obtusa* in North America (Larkin et al., 2018), and the red alga *Bangia atropurpurea* in North America (Kwei & John, 1977; Shea et al., 2014). The freshwater red alga *Montagnia macrospora* (Batrachospermales, Rhodophyta) is a recently reported introduced case (Kato et al., 2009). This alga is native to the tropical areas of South America, spanning French Guiana, Bolivia, and Brazil (Necchi et al., 2019; Vis et al., 2008). Instances of *M*. *macrospora* in East Asia were first reported from a stream in Taoyuan in Taiwan in 2005 (Chou et al., 2014) and from an artificial pond in Okinawa, Japan in 2006 (Kato et al., 2009). In 2014, *M*. *macrospora* was found in multiple locations in Malaysia (Johnston et al., 2014). Recently, Zhan et al. (2021) found *M*. *macrospora* in many aquarium shops across East Asia, including Hat Ya (southern Thailand, Malay Peninsula), Hong Kong, Taiwan, and Okinawa (Japan). A genetic analysis based on the plastid marker *rbc*L has suggested that there were at least two independent introductions of *M*. *macrospora* into East Asia (Zhan et al., 2021). One of the introductions was revealed by a haplotype in aquarium shops in Taiwan and Japan, and the other introduction by a different haplotype in aquarium shops in Hong Kong and Thailand (Zhan et al., 2021). Thus, Zhan et al. (2021) proposed that *M*. *macrospora* was introduced into East Asia via the global aquarium trade. Although not traded for ornamental purposes, *M*. *macrospora* can hitchhike on ornamental organisms (e.g., aquatic plants), which are traded between South America and Taiwan (Zhan et al., 2021).

There is no consensus view on how to determine whether an introduced species has become invasive. Often, researchers recognize an invasive species when it has already exerted noticeable ecological or socioeconomic impacts (Pyšek et al., 2008). However, it has been argued that such a strict definition would prevent the early detection of invasive species and might hinder their conservation and management (Pyšek et al., 2008). From a conservation planning and management perspective, it is critically important to identify the invasive species at an early stage, particularly before it exerts any noticeable ecological and/or socioeconomic impact. Richardson and Pyšek (2012), therefore, argued that the term ‘invasive’ should not imply environmental impact and that a successful invasion simply means an invasive species has become self‐sustaining and widespread populations across a given area. Specifically, introduced species can become naturalized (a self‐sustaining founder population, but not spreading) in a non‐native range and then may become invasive (overpopulated and/or widespread), potentially making adverse ecological impacts (e.g., wiping out native species and disrupting invaded ecosystems) and socioeconomic impacts (e.g., incurring conservation management costs) (reviewed in Richardson & Pyšek, 2012; Richardson et al., 2000).

To assess the invasive potential of *M*. *macrospora*, it is crucial to understand its basic ecology. It is unknown whether *M*. *macrospora* is ecologically a generalist or specialist. Invasive species are typically generalists that are ecologically competent, and can establish and spread their populations under a wide range of environmental conditions (reviewed in Gioria & Osborne, 2014). For example, introduced macroalgae can grow well in polluted waters with a high amount of certain nutrients (e.g., nitrate and phosphate), whereas native algae can typically grow well only in clean waters (Inderjit et al., 2006).

**FIGURE 1:**
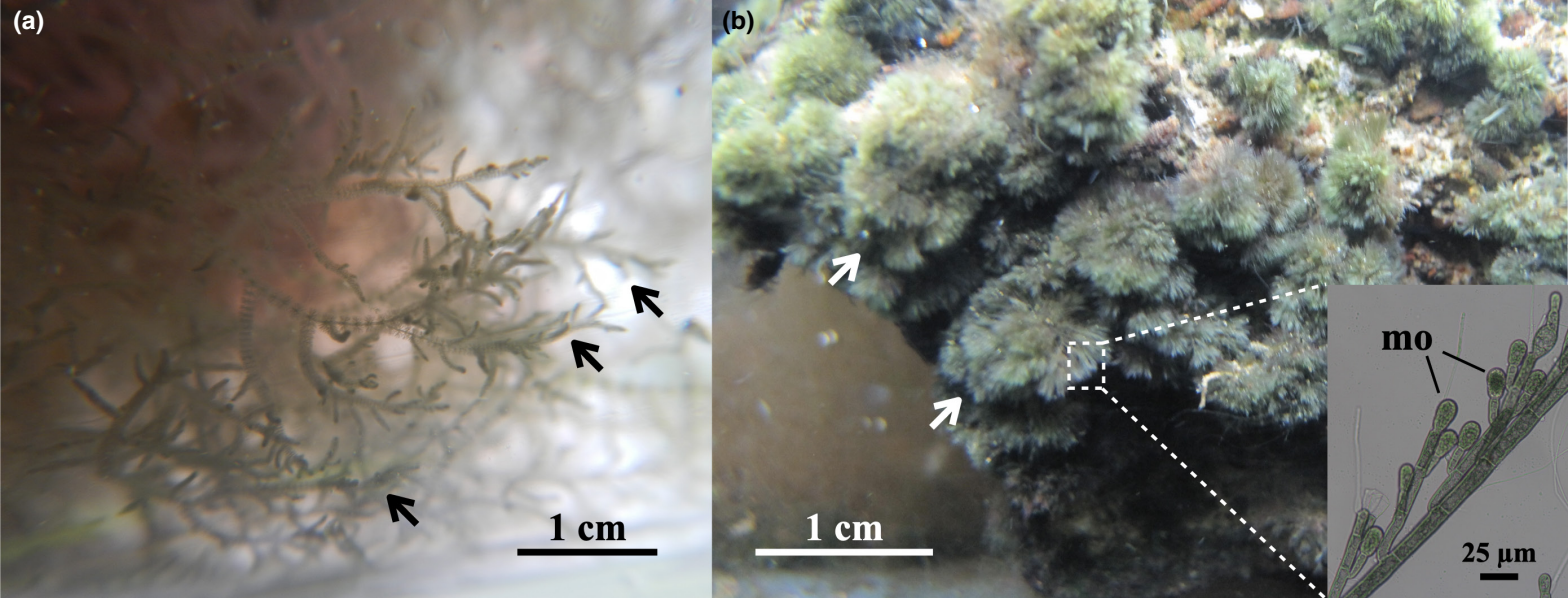
In situ photos of *Montagnia macrospora*. (a) A gametophyte (black arrows) collected from Ma‐Tai'an wetland, Hualien, in eastern Taiwan. (b) Chantransia (i.e., sporophytes) (white arrows) from the Taoyuan stream, Taiwan. The inset indicates the close‐up microscopic view of asexually reproductive structures—monospores (mo)

Propagation via asexual spores or vegetative fragmentation is a fast and efficient way to spread in the absence of available mating partners. This ability can facilitate an alien species to self‐sustain and thus become naturalized in and invade non‐native ranges. *M*. *macrospora* undergoes alternation between two free‐living heteromorphic generations. As a haploid gametophyte (Figure 1a), the alga takes on a sexual thread‐like mucilaginous form that produces and releases gametes (Necchi et al., 2019). As a turf‐like diploid sporophyte (also known as chantransia; Figure 1b), the alga produces mostly asexual monospores and vegetative fragments (which can attach to other aquatic organisms and surfaces) and rarely sexual meiospores (Kato et al., 2009). The specimens of *M*. *macrospora* previously observed in a survey of aquaria and streams in Taiwan were mostly spore‐producing chantransia (Zhan et al., 2021).

In this study, we took a multifaceted approach to determine whether *M*. *macrospora* has become an invasive species in Taiwan and to examine the traits that might have facilitated its invasion. First, we assessed the extent of its spread across Taiwan via field surveys. Second, to identify the main climatic components that differ between the alga's native range in South America and its non‐native range in Taiwan, we compared the climates of the two geographic regions. Third, we determined the population structure of *M*. *macrospora* in South America and East Asia by reconstructing a haplotype network based on the intergenic spacer between the cytochrome oxidase subunit 2 and subunit 3 genes (*cox2*‐*3*), which is a widely used marker to evaluate interpopulation genetic variation in freshwater red algae (Necchi & Vis, 2005; Paiano & Necchi, 2013, 2017). Fourth, we measured pH and nutrient level of freshwater bodies around Taiwan where *M*. *macrospora* has been found. Last, we performed a monthly ecological survey of a stream in Taoyuan, where Chou et al. (2014) reported the presence of *M*. *macrospora* in Taiwan for the first time in 2005. We collected data about the percent cover of *M*. *macrospora*, occurrences of other macrophytes, and water quality of the stream. This study provides a multifaceted assessment of the ecological status of *M*. *macrospora* as an invasive freshwater macroalga in Taiwan. Filling this knowledge gap may be important for the conservation planning and management of this alga.

## 2 MATERIALS AND METHODS

### 2.1 Field inspections and environmental measurements

**FIGURE 2:**
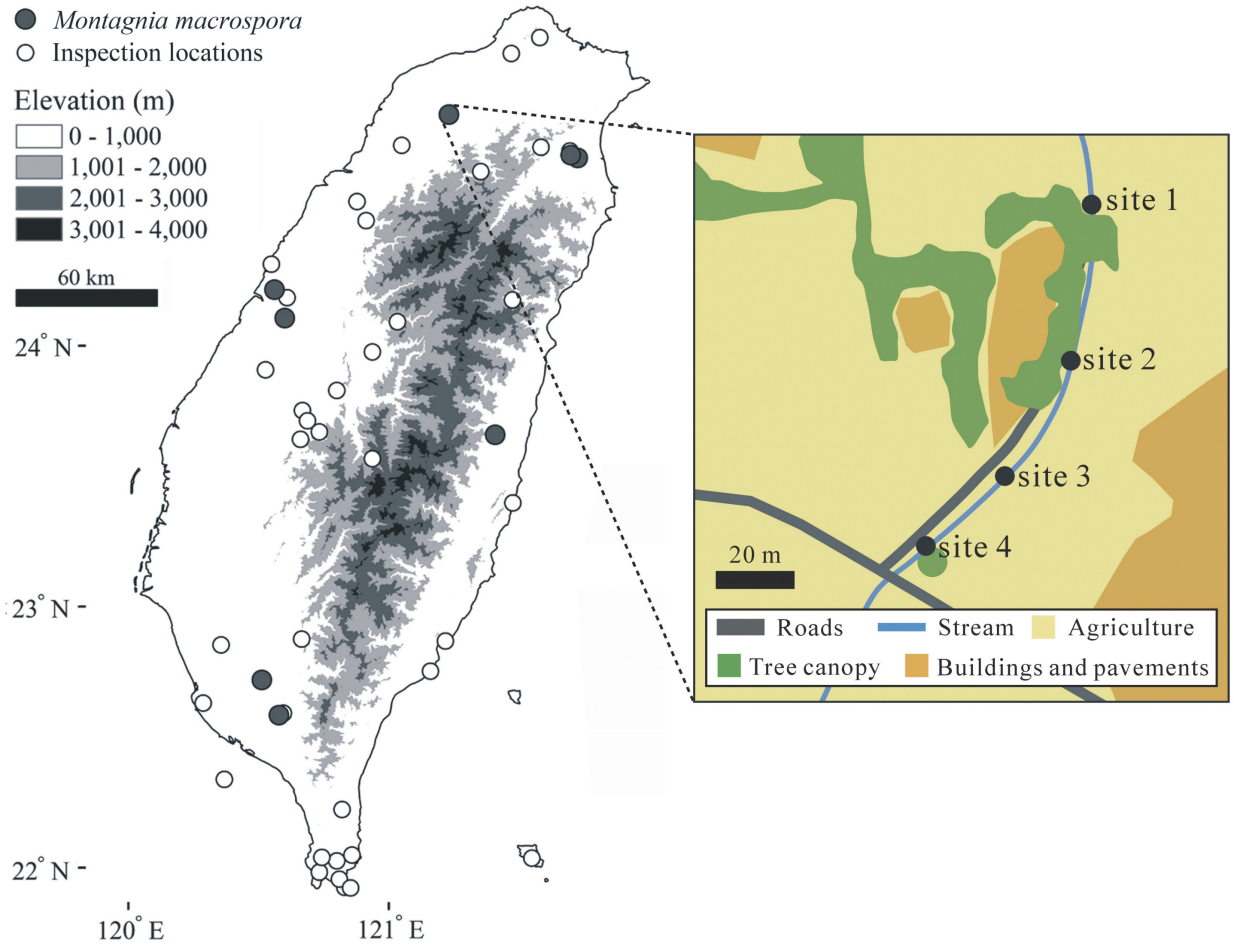
Locations inspected for the presence of *Montagnia macrospora* in Taiwan. The gray circles represent the locations where *M*. *macrospora* was found, while the white circles represent the locations where *M*. *macrospora* was not found (as inspection locations). Inset map showing the monthly surveys at four sites, from site 1 (downstream) to site 4 (upstream), along the Taoyuan stream (24°53′09.3″N, 121°13′54.1″E), and surrounding land use

To assess the distribution of *M*. *macrospora* in Taiwan, we carried out field inspections at 47 locations. *M*. *macrospora* was found in eight locations (Figure 2). We measured the pH and nutrient levels at the eight locations and in the artificial reservoir pond in Okinawa (Japan), where the presence of *M*. *macrospora* was first reported in East Asia (Kato et al., 2009). The pH and nutrient measurements were mostly a one‐time measure, except for two locations: the Taoyuan stream and the Taichung Industrial Park Outlet. A seasonal survey was carried out at the Taoyuan stream (see hereafter). The Taichung Industrial Park Outlet was surveyed three times, in January 2014 and in July and September 2021. *M*. *macrospora* was observed each time, and measurements were obtained in January 2014 and July 2021. The pH was measured using a pH meter (PH30, CLEAN Instruments Co., Taiwan). For nutrient measurements, water samples of 1000 ml were collected and stored in an ice box during transportation. Then, measurements of four nutrients (ammonia, nitrate, nitrite, and phosphate, in mg L^−1^) were taken in our laboratory using a SMARTSpectro spectrophotometer (LaMotte).

### 2.2 Climatic differences between the native and non‐native ranges of *Montagnia macrospora*

To examine the climatic difference of *M*. *macrospora* between its native range in South America and its non‐native ranges in Taiwan, we compared the climate of the 16 sampling locations of *M*. *macrospora* in South America surveyed in previous studies (Chiasson et al., 2005; Necchi & Oliveira, 2011; Vis et al., 2008; Vis & Sheath, 1999) and the eight locations in Taiwan. The current climatic CMIP5 data, which includes 19 bioclimatic variables and elevation with a spatial resolution of 2.5 arc minutes, were obtained from the WorldClim database (http://www.worldclim.org/). To exclude highly correlated and redundant variables, we conducted a multicollinearity test using the variance inflation factor (VIF) using the R package *usdm* version 1.1‐18 (Naimi et al., 2014). The following ten predictors, which had a VIF score of at least 10, were used in downstream analyses (Table S1): elevation, mean diurnal range (bio02), isothermality (= mean diurnal range/temperature annual range * 100; bio03), mean temperature of wettest quarter (bio08), mean temperature of driest quarter (bio09), precipitation of wettest month (bio13), precipitation of driest month (bio14), precipitation seasonality (coefficient of variation; bio15), precipitation of warmest quarter (bio18), and precipitation of coldest quarter (bio19). The correlation between each variable did not exceed the recommended threshold of 0.75 (Kumar et al., 2006; Figure S1). To identify the main climatic factors differentiating South America and Taiwan, we normalized the ten variables using the z‐score approach and then applied a principal component analysis (PCA) using the R package *Stats* version 3.6.0 (R Core Team, 2020). The field inspection locations of *M*. *macrospora* in Taiwan were marked on a map using ArcGIS version 10.1 (ESRI).

### 2.3 Population genetic analysis

Thirty specimens of *M*. *macrospora* were collected from aquaria and field locations (e.g., streams and springs) in Taiwan (Table S2). Two additional specimens were collected from Okinawa, where *M*. *macrospora* was also found introduced (Kato et al., 2009). A portion of each specimen (100–200 mg) was preserved in either silica gel or 95% ethanol for DNA sequence analysis. Another portion of each specimen was suspended in a 10%–15% formalin solution for morphological identification. Total genomic DNA was extracted using the ZR Plant/Seed DNA kit (Zymo Research), following the manufacturer's recommendations. The genetic marker *cox2*‐*3* was amplified using the primer pair: COX2F (5′‐GTACCWTCTTTDRGRRKDAAATGTGATGC‐3′) and COX3R (5′‐GGATCTACWAGATGRAAWGGATGT C‐3′) under the PCR conditions described in Vis et al. (2008). Sanger sequencing of PCR products was subsequently performed by the Mission Biotech Company (Taipei, Taiwan). As there was a lack of nucleotide diversity in the newly generated *cox2*‐*3* sequences, a single representative has been deposited in NCBI GenBank (accession: OK442460).

For a population genetic analysis, we retrieved eight additional *cox2*‐*3* sequences of *M*. *macrospora* specimens found in the native range of the alga in South America from GenBank (Table S2). The combined sequence set, which included our newly generated sequences and the sequences retrieved from GenBank, was aligned using MUSCLE (Edgar, 2004) via MEGA X version 10.1.7 (Kumar et al., 2018). A haplotype network was inferred from the multiple sequence alignment using the TCS method (Clement et al., 2000), which uses the statistical parsimony approach implemented in PopART version 1.7 (Leigh & Bryant, 2015).

### 2.4 Ecological surveys of a stream in Taoyuan, Taiwan

Field surveys between August 2012 and October 2013 were carried out at four sites along a small spring‐fed stream in Longtang District, Taoyuan City, Taiwan (24°53′09.3″N, 121°13′54.1″E). We conducted the surveys once a month except in December 2012 due to logistic issues and weather conditions. The four sites were 10 m apart from each other, going from downstream (site 1) to upstream (site 4) (Figure 2; Figure S2). The stream runs adjacent to a small alley in a flat farm area. During our surveys, we observed that the bed of the stream was covered with mud and silt, and artificial substrates (cement, gravel, and some sandbags).

The four sites showed variation in vegetation and surrounding structures, which caused differences in illumination. Site 3 was most exposed to sunlight, and the other three sites showed different degrees of tree canopy shading. Site 1 was muddier than the other sites, sites 2 and 3 had cemented stream beds, and site 4 had silt in its bed. To calculate the percent cover of *M*. *macrospora* at each site, a transect was placed underwater across the width of the stream bed (i.e., perpendicular to the flow) (the width ranged between 50 to 380 cm), then five 10 × 10 cm quadrats were positioned along the transect at equal distance. The distance between quadrats varied depending on the total length of the transect. The percent cover of the algae was calculated as the average of the percent cover of the five quadrats of the site. *M*. *macrospora* was identified in situ based on the color (blue‐green) and the overall external morphology of the thallus. To further confirm the species identity of the algae, five specimens per site were randomly collected from the quadrats and brought back to the laboratory. This was followed by *cox2*‐*3* molecular verification for random samples as a training process for confirming our morphological identification. Eleven of these sequences were utilized for the population genetic analysis (see Section 2.3 and Table S2), while the others were discarded as they were all identical (data not shown). During each monthly survey, light intensity (µmol photons m^−2^ s^−1^), water temperature (ºC), pH, conductivity (µS cm^−1^), turbidity (mg L^−1^), water velocity (m s^−1^), and water depth (cm) were measured at each of the four sites by using a portable digital photometer (DL‐204 EZDO, Chi‐Jui Instrument Enterprise), a thermometer, a pH meter (PH30, CLEAN Instruments Co.), a meter for both conductivity and turbidity (CON30, CLEAN Instruments Co.), a flow probe (FP111 Global Water, Smart Scientific Corp.), and a ruler, respectively. The levels of four nutrients (i.e., ammonia, nitrate, nitrite, and phosphate) were measured as described above. Precipitation data at Zhongli automatic weather station, located in Zhongli District, Taoyuan City was retrieved from the Central Weather Bureau, Taiwan (https://www.cwb.gov.tw), and hours of daylight were retrieved from the National Oceanic and Atmospheric Administration (NOAA) (https://gml.noaa.gov/grad/solcalc/).

We examined the variation in the percent cover of *M*. *macrospora* and environmental variables among the four sites and determined which environmental variable was significantly correlated with the percent cover for each site. First, to determine whether there was any variation in the percent cover of *M*. *macrospora* and environmental variables among the four survey sites, we applied a one‐way Analysis of Variance (ANOVA), followed by multiple comparisons among each pair using a post hoc Tukey's test if significant effects were detected. Prior to ANOVA, the light intensity of each month was normalized among the four sites based on the relative light percent difference (i.e., corresponding to different degrees of light shading by tree canopy). Second, to examine the effects of the environmental variables on the percent cover of *M*. *macrospora* at each site, we applied multiple regression analyses. Ten environmental variables were included in the analysis: water temperature, pH, conductivity, turbidity, ammonia, phosphate, nitrite, nitrate, accumulated precipitation one week before collection, and day length. Prior to the regression analysis, the ten variables were standardized as z‐scores. We did not include light intensity, water velocity, and water depth, because they were affected by rain clouds and rainfall during or before our field work. If significant effects were detected, we additionally calculate the Pearson correlation coefficients among the ten selected environmental variables, as some of them had the potential to be highly correlated to each other (i.e., confounding factors). All the statistical analyses were performed in R (R Core Team, 2020).

### 2.5 Co‐occurring, non‐native aquarium‐associated macrophytes

During each survey in the Taoyuan stream, we recorded the presence of other aquarium‐associated freshwater red algae and introduced aquatic plants that are commonly found in aquarium shops. Species identification of aquatic plants was performed based on several guidebooks (Lin, 2009a, 2009b, 2009c). Species identification of freshwater red algae was done based on the sequences of the *rbc*L gene.

## 3 RESULTS

### 3.1 Occurrences across Taiwan and climatic differences between Taiwan and South America

**FIGURE 3:**
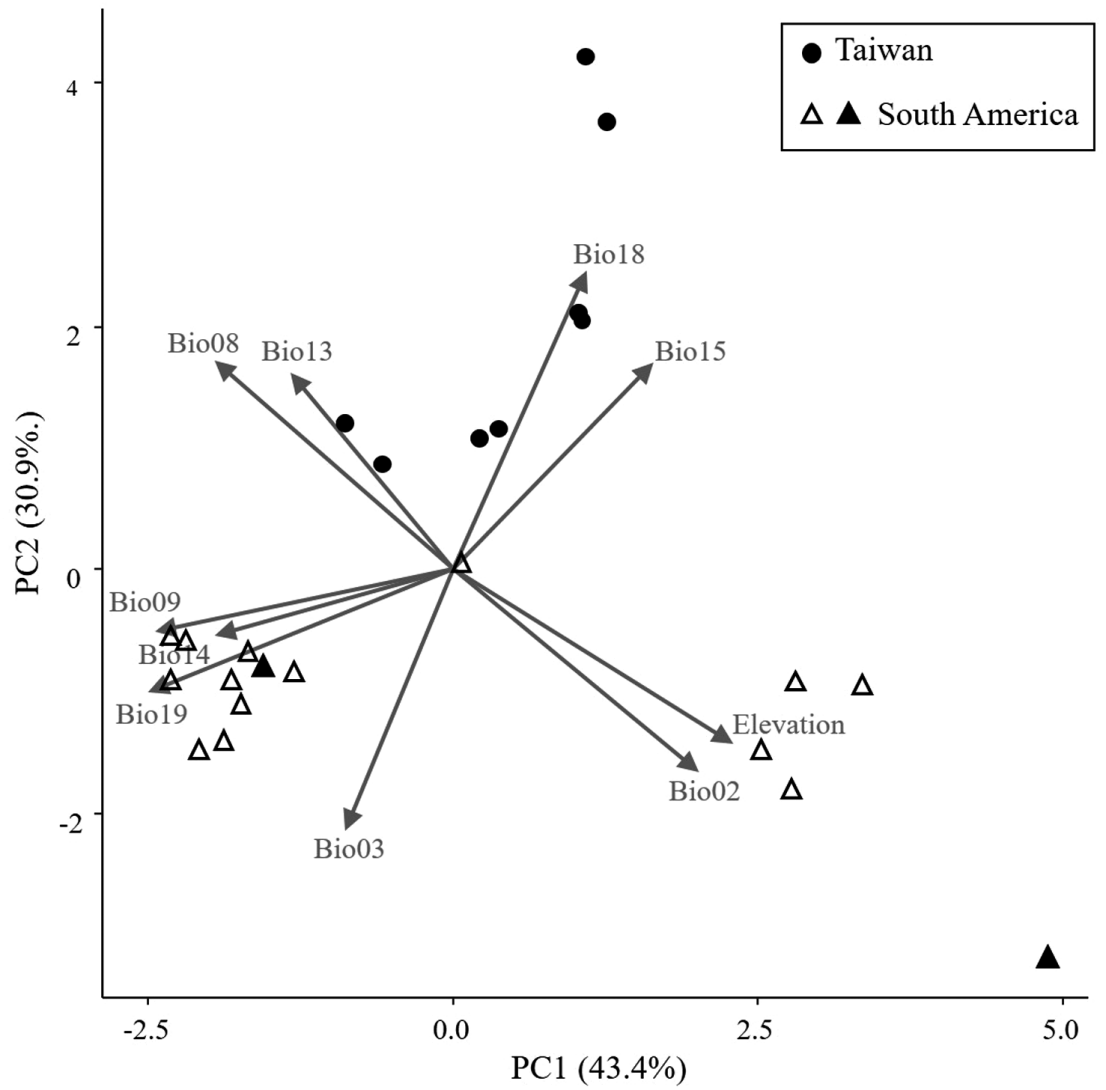
A principal component analysis showing the climatic differences between the non‐native range of *Montagnia macrospora* in Taiwan and the native range of *M*. *macrospora* in South America. Arrows indicate the degree of correlation between ten variables and two principal components (PC1 and PC2). The black circles represent the locations in Taiwan, while the white triangles represent the locations in South America

Our island‐wide inspections revealed the presence of *M*. *macrospora* in eight out of 47 (~17%) locations in Taiwan (Figure 2, Table S2). A PCA based on ten climatic variables indicated that the first two components (PCs) of the PCA accounted for 74.3% of the total variance of the data (PC1: 43.4% and PC2: 30.9%; Figure 3). The locations in Taiwan and in South America were primarily differentiated by PC2 (Figure 3). We took the absolute value of 0.3 as the load threshold to determine which variable substantially contributed to PC2. The variables mean diurnal range (bio02) and isothermality (bio03) had higher values in Taiwan compared to South America (negatively loaded), whereas the variables mean temperature of wettest quarter (bio08), precipitation of wettest month (bio13), precipitation seasonality (bio15), and precipitation of warmest quarter (bio18) had lower values in Taiwan compared to South America (positively loaded) (Table S3).

### 3.2 Lack of nucleotide diversity in *cox2*‐*3* in *Montagnia macrospora*

**FIGURE 4:**
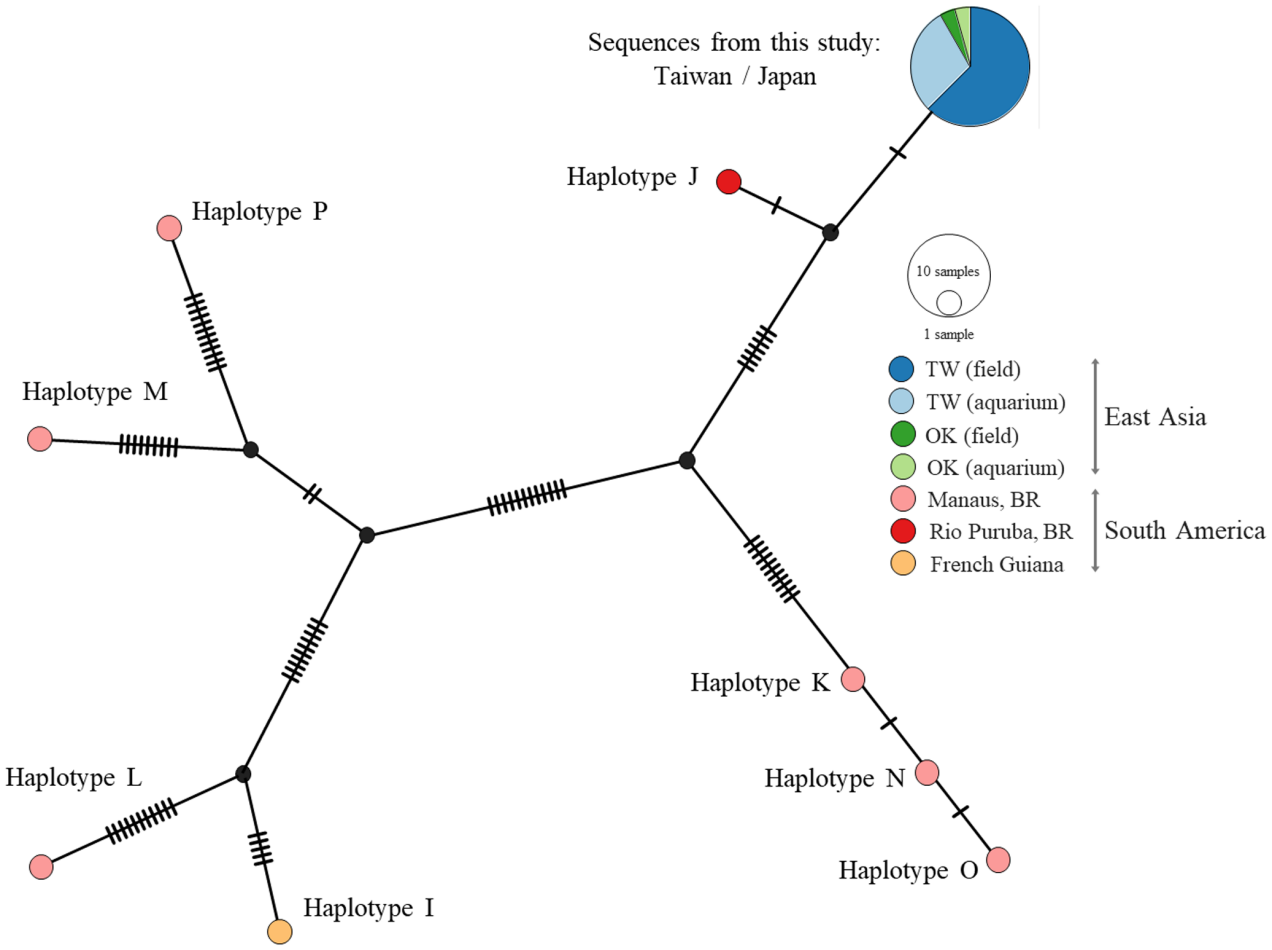
A *cox2*‐*3* haplotype network of *Montagnia macrospora* inferred using the TCS method. The hatch marks on the links between the haplotypes represent nucleotide differences between the two haplotypes. One haplotype represents the sequences obtained from this study (from Taiwan and Japan) and the other eight haplotypes belong to those from South America (the sequences were retrieved from NCBI Genbank, keeping their names as in the original reference). The samples from South America show higher genetic diversity. None of the sequences from South America share the same haplotypes with the samples obtained from this study. Haplotype J, however, is the closest, with only two mutational steps of separation from the haplotype found in Taiwan and Japan. Abbreviation: TW, Taiwan; OK, Okinawa; BR, Brazil

The *cox2*‐*3* sequence alignment from 40 specimens of *M*. *macrospora* was 338 bp in length. There was a 4‐bp gap in all the sequences from Taiwan and Japan and in five out of the eight haplotypes from South America. The haplotype network analysis indicated identical *cox2*‐*3* sequences in all the specimens collected from both aquaria and fields in Taiwan and Japan (Figure 4). In contrast, higher genetic diversity was found in the specimens of the algae from the native range in South America, consistent with the pattern of the *rbc*L haplotype diversity shown in Zhan et al. (2021).

### 3.3 Presence of *Montagnia macrospora* in Taiwan in a wide range of pH and nutrient levels

**FIGURE 5:**
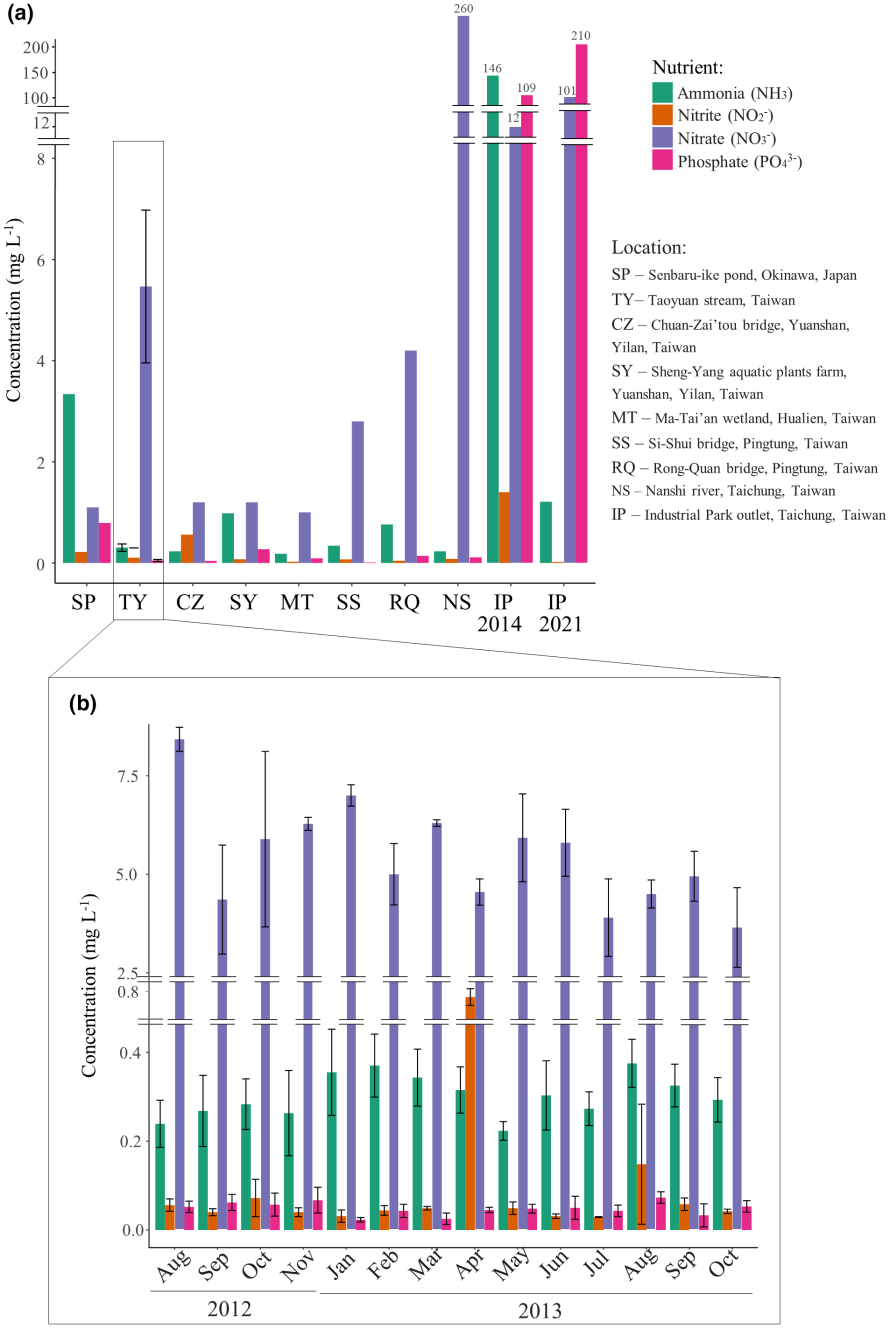
Concentration of four main nutrients: ammonia, phosphate, nitrite, and nitrate. (a) Nutrient concentration in streams and ponds in Taiwan and Japan where *Montagnia macrospora* was found. For the Taoyuan stream, where monthly surveys were performed, the error bars represent standard deviations based on all measurements taken during the survey. For all the other locations, the level of nutrients was measured once and, therefore, error bars cannot be computed. (b) Monthly nutrient concentrations in the Taoyuan stream. The error bars represent standard deviations based on four separated sites in the Taoyuan stream

We examined the pH and nutrient levels of the eight locations in Taiwan and the one in Okinawa where *M*. *macrospora* was found. A broad range of pH was observed across the sites (5.67–8.34). Similarly, the nutrient levels varied widely across the sites in Taiwan. The levels of ammonia ranged from 0.18 to 0.76 mg L^−1^; Taichung Industrial Park Outlet was an outlier, with a level of ammonia of 146 mg L^−1^ in 2014 (at least 150 times higher than the other locations) that decreased to 1.21 mg L^−1^ in 2021. The nitrite levels ranged from 0.02 mg L^−1^ to 1.4 mg L^−1^. The nitrate levels ranged from 1 mg L^−1^ to 6 mg L^−1^; Taichung Industrial Park Outlet and Nanshi river were outliers, with 101 mg L^−1^ and 260 mg L^−1^ in 2014 (at least 20 and 50 times higher than the other locations), respectively. The phosphate levels were low, ranging from 0.01 to 0.27 mg L^−1^; again, Taichung Industrial Park Outlet was an outlier with 109 mg L^−1^ in 2014 and 210 mg L^−1^ in 2021, at least 400 times higher than the other locations (Figure 5). Kato et al. (2009) reported that the artificial reservoir pond in the Senbaru‐ike in Okinawa, Japan, where *M*. *macrospora* was found, was “very eutrophic” but did not provide nutrient measurements. The presence of *M*. *macrospora* was visually confirmed in the same pond in 2013, and our nutrient measurement showed a higher level of ammonia (3.34 mg L^−1^) compared to most of the sites in Taiwan, while the level of nitrate (1.1 mg L^−1^), nitrite (0.22 mg L^−1^), and phosphate (0.79 mg L^−1^) were lower (Figure 5).

### 3.4 Ecological preferences of *Montagnia macrospora* in Taiwan

**FIGURE 6:**
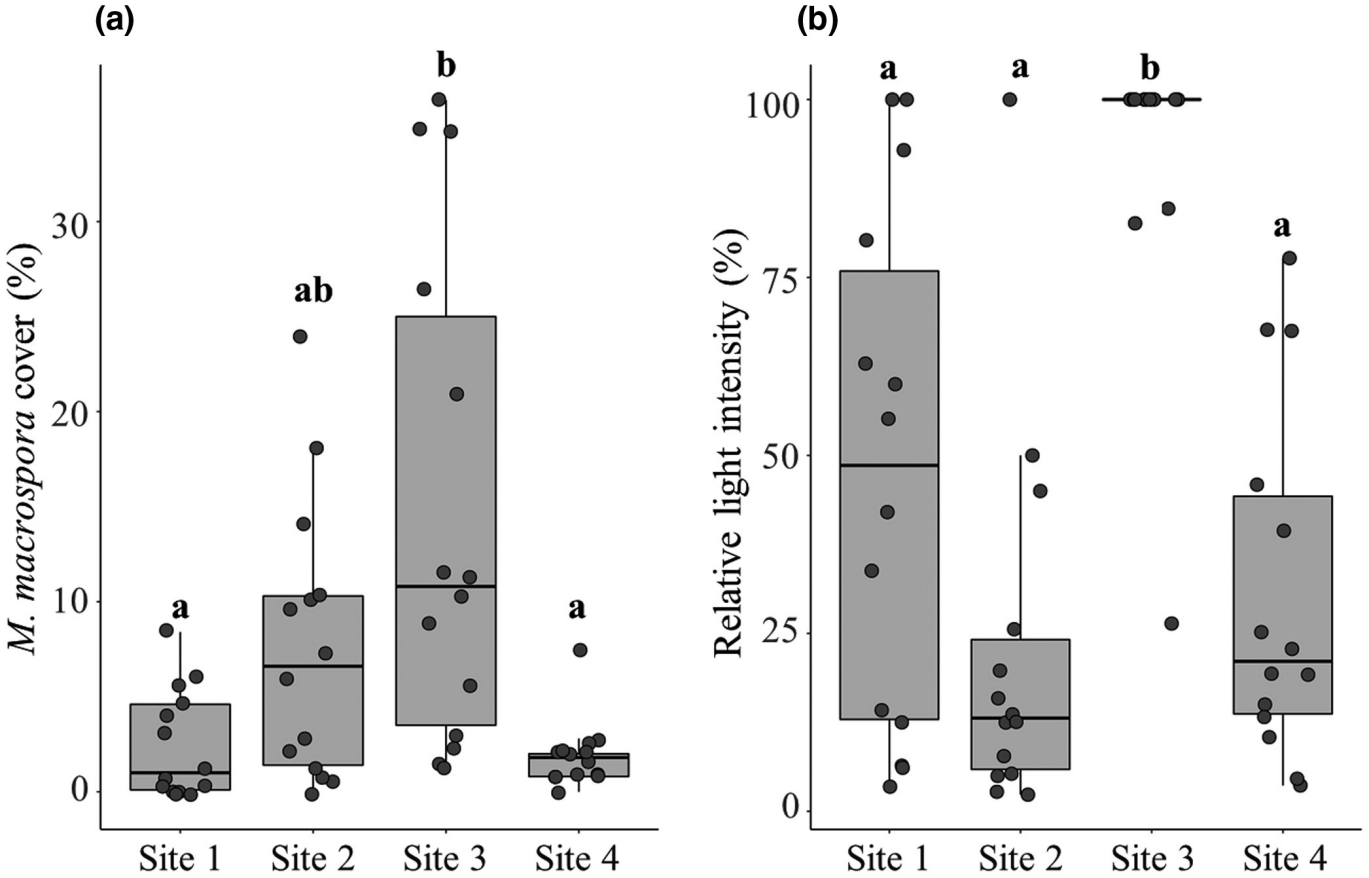
Differences in the percent cover of *Montagnia macrospora* (a) and relative light intensity (b) among four surveyed sites along the Taoyuan stream. Of all the parameters measured, the percent cover of *M*. *macrospora* and relative light intensity were the only two variables that showed significant variation among the four sites (one‐way ANOVA, Table S4). *Post hoc* Tukey's tests showed significant variation between site 3 and the other three sites, with site 3 having both a higher percent cover of *M*. *macrospora* and relative light intensity. For each site, single observations are represented as jittered points (*n* = 14)

Over our 14‐month field surveys in the Taoyuan stream, of all the parameters measured, the percent cover of *M*. *macrospora* and relative light intensity were the only two variables that showed significant variation among the four sites (one‐way ANOVA, Table S4). Post hoc Tukey's tests showed the percent cover of *M*. *macrospora* at site 3 was significantly higher than the percent cover at site 1 and site 4 (*p* < .001; ANOVA and *post hoc* Tukey's test; Figure 6a; Table S5). The mean percent cover was higher at site 3 compared to site 2; however, the difference between these two sites was not statistically significant (*p* = .0699, ANOVA and post hoc Tukey's test; Figure 6a; Table S5). Relative light intensity was significantly higher at site 3 than at the other three sites (*p* < .001; one‐way ANOVA) (Figure 6b; Table S6). Over the course of our field surveys, the most shaded sites (site 2 and site 4) had a lower light intensity (2 to 2500 µmol photons m^−2^ s^−1^), and a less shaded site (site 1) had higher light intensity (3 to 8000 µmol photons m^−2^ s^−1^). In contrast, site 3 was a well‐lit, open site that had the highest light intensity (45–19,000 µmol photons m^−2^ s^−1^).

**FIGURE 7:**
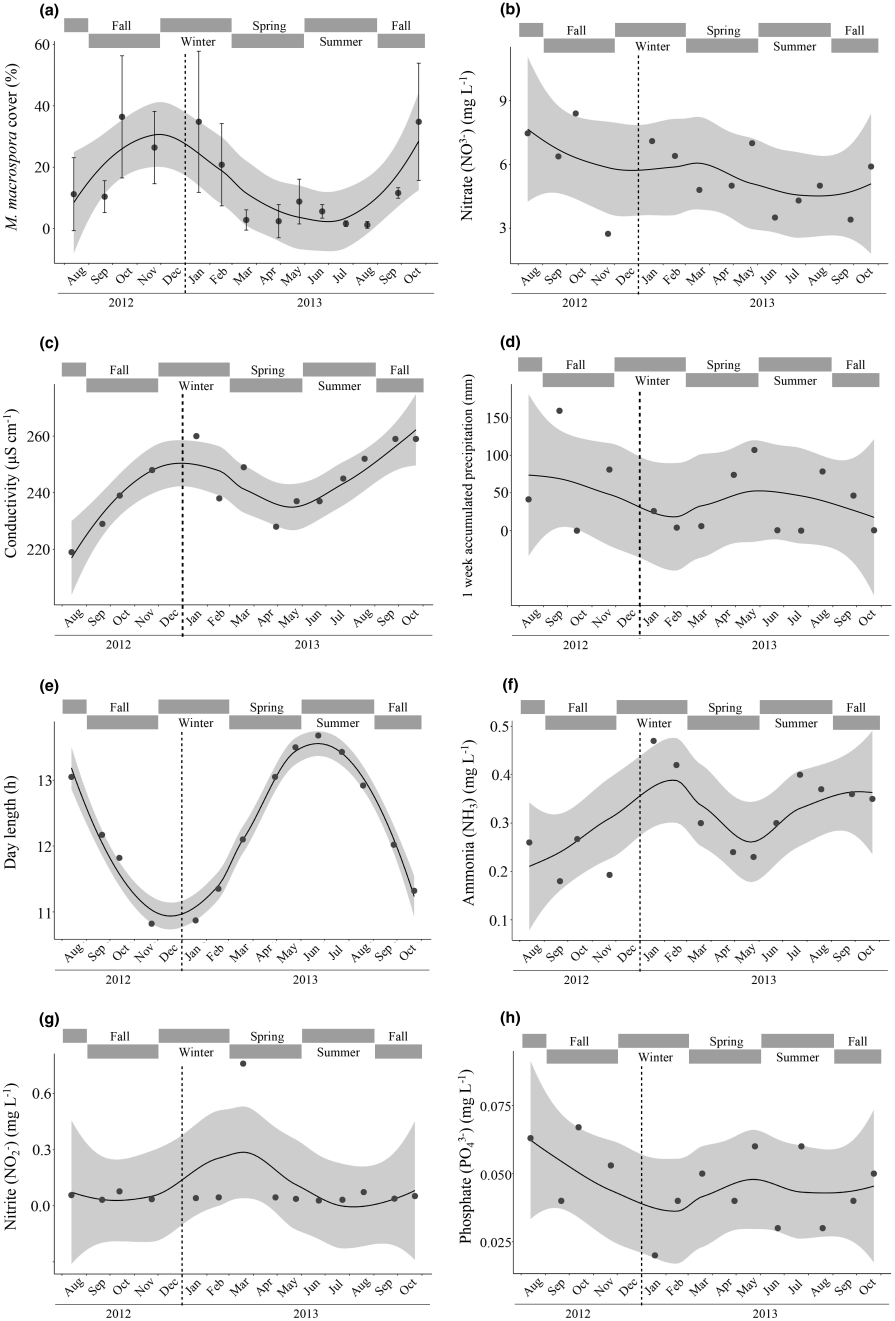
Curve fitting showing the seasonal pattern of percent cover of *Montagnia macrospora* (a) and environmental variables (b–h) at site 3 during a 14‐month survey in the Taoyuan stream. Only environmental variables that are significantly correlated with the change in the algal cover based on multiple regression analyses with *p* < .05 are shown as follows: nitrate level (b), conductivity (c), the amount of precipitation accumulated one week before each survey (d), day length (e), ammonia (f), nitrite (g), and phosphate levels (h). The error bars represent standard deviations based on five quadrats along the transect. The shaded area represents a 95% confidence interval around the smoothed regression line. Seasons are indicated as gray bars above each plot

During the surveys, the algal cover ranged from nearly 0% to 18% at sites 1, 2, and 4, with no clear seasonal pattern (Figure S3 and Tables S7–S9). However, the percent cover of *M*. *macrospora* at site 3 followed a seasonal pattern. It was significantly correlated with day length and went as high as 36.4% during the winter when the days were shorter (10 h 49′) and the water temperature was lower (e.g., 16.7°C), and it went as low as 1.2% during the summer when the days were ~3 h longer and the water temperature was higher (up to 27.3°C) (Figure 7a; Figure S3).

At sites 1, 2, and 4, none of the environmental variables were significantly correlated with the temporal change of the percent cover of the algae (Tables S7–S9). At site 3, the percent cover of *M*. *macrospora* (Figure 7a) was positively correlated with nitrate and water conductivity (a proxy for the concentration of ions) (Figure 7b,c; Table S10), but it was negatively correlated with precipitation that accumulated for one week before the survey, day length, ammonia, nitrite, and phosphate (Figure 7d–h; Table S10). The Pearson correlation analysis (presented only for site 3, Table S11) revealed a strong positive correlation between two pairs of environmental variables: day length and water temperature, and conductivity and turbidity. Water temperature and conductivity did not show a significant correlation with algal growth because of their multicollinearity with day length and turbidity, respectively. Further analysis and experiments will be necessary to establish which of the collinear factors mostly influences algal growth. Although we found the presence of other macrophytes (see below), *M*. *macrospora* was the most dominant species in the periphyton communities in the stream (for example, see Figure 1b).

In addition to the field surveys in the Taoyuan stream, we found *M*. *macrospora* in the Taichung Industrial Park Outlet in January 2014 and then in July and September 2021 (a span of nearly eight years). We observed that *M*. *macrospora* bloomed in 2021, its percent cover exceeded 50% of all available substrates, such as bamboo shoots, grass shoots, and fishing nets (Figure S4). This site is warm (25.8°C in winter, to 29.7°C in fall), acidic (pH 5.67–6.21), and highly eutrophic (see Section 3.3 for details).

### 3.5 Observations of other aquarium‐associated macrophytes

During the monthly surveys of *M*. *macrospora* in the Taoyuan stream, we made observations of several macrophytes that are commonly sold in aquaria (Table 1, Figure S5). Many of the macrophytes were aquatic plants that are commonly transported via the freshwater aquarium trade (*Egeria densa*, *Riccia fluitans*, *Rotala rotundifolia*, and *Vesicularia dubyana*). Besides *V*. *dubyana*, these species are not native to Taiwan or Asia. Also, we found three freshwater red algae that are commonly present in aquaria: *Compsopogon caeruleus*, *Nemalionopsis shawii*, and *Sheathia dispersa* (Table 1).

**TABLE 1:** List of aquarium‐associated macrophytes co‐occurring with *Montagnia macrospora* in the Taoyuan stream. These species are commonly found in aquarium shops. Their in situ photos are shown in Figure S5

Species name	Notes	Reference
Aquatic plants		
*Egeria densa*	Native to South America. An invasive species spread via the aquarium trade. Common commercial ornamental plant in freshwater aquarium shops.	Yarrow et al. (2009)
*Riccia fluitans*	Native to North America. Common commercial ornamental plant in freshwater aquarium shops.	Center for Aquatic and Invasive Plants, University of Florida (https://plants.ifas.ufl.edu/plant‐directory/riccia‐fluitans/)
*Rotala rotundifolia*	Native to Asia (including India, Japan, and China). An invasive species in the United States. Commercial ornamental plant in freshwater aquarium shops.	Burks et al. (2003)
*Vesicularia dubyana*	Native to Southeast Asia. Commercial ornamental plants in freshwater aquarium shops.	Goffinet et al. (2009)
Freshwater red algae		
*Compsopogon caeruleus*	Cosmopolitan. Commonly found in aquarium shops.	Stoyneva et al. (2006), Zhan et al. (2021)
*Nemalionopsis shawii*	Native to East Asia. Occasionally found in aquarium shops.	Zhan et al. (2021)
*Sheathia dispersa*	Cosmopolitan. Frequently found in aquarium shops.	Zhan et al. (2021)

## 4 DISCUSSION

*Montagnia macrospora* is a freshwater red macroalga commonly found in South America (Necchi et al., 2019). It is not until recently that the global aquarium trade was uncovered to be the source of its introduction throughout East Asia (Kato et al., 2009; Zhan et al., 2021). In Taiwan, the earliest record of this alga in the field is dated back to 2005 (Chou et al., 2014). In this study, we conducted a multifaceted ecological assessment of *M*. *macrospora* in Taiwan via a combination of field surveys, population genetic analysis, and longitudinal environmental monitoring.

Our island‐wide survey revealed that *M*. *macrospora* is widespread in the field across Taiwan (about 17% of the locations inspected). In our monthly survey of the Taoyuan stream, *M*. *macrospora* was found with other aquarium‐associated macrophytes, indicating that the alga is likely released into the environment via the disposal of aquarium water and/or unwanted aquatic plants. This observation is consistent with the findings by Zhan et al. (2021), which show that *M*. *macrospora* was commonly found in aquarium shops in Taiwan.

Zhan et al. (2021) have proposed that aquarium shops and aquatic plant farms may serve as a source of introduction of *M*. *macrospora* in the field. The data from Zhan et al. (2021) and this study revealed that the sequences of *rbc*L and *cox2*‐*3* are identical across the populations of *M*. *macrospora* in Taiwan and Okinawa (Japan). This result is consistent with the hypothesis that a founder population of *M*. *macrospora* from the native range was introduced in a single event. Then, its spread was probably followed by rapid clonal expansion via asexual reproduction among the local aquarium shops or farms, or by secondary dispersal via waterfowl or rats as viable algae can be found on their feet or in their gut (Sheath & Hambrook, 1990).

We observed that *M*. *macrospora*, once introduced into new environments, can self‐sustain for years. *M*. *macrospora* was observed in the Taoyuan stream from 2005 to 2013 (~nine years); in the Senbaru‐ike Pond, Okinawa from 2006 to 2013 (~eight years); and in the Taichung Industrial Park Outlet from 2014 to 2021 (~eight years) (this study; Chou et al., 2014; Kato et al., 2009). In the Taichung Industrial Park Outlet, blooming was observed in 2021. These observations suggest that *M*. *macrospora* has become naturalized in Taiwan, self‐sustaining in the same sites without human intervention. Overall, these observations, combined with population genetic analyses, suggest a single introduction event followed by naturalization and island‐wide secondary dispersal for *M*. *macrospora*, indicating that *M*. *macrospora* has become invasive in Taiwan based on the definition suggested by Richardson and Pyšek (2012).

Studies have shown that species of freshwater red algae usually are not ecologically generalists. Most species of freshwater red algae exhibit a narrow range of environmental preferences, for example, dim light conditions, moderate temperature fluctuations, certain acidity, and clean waters (Carmona et al., 2011; Eloranta et al., 2016; Evans & Vis, 2020; Sheath & Hambrook, 1990; Sheath & Vis, 2015). However, several lines of evidence from this study indicated that *M*. *macrospora* is an ecological generalist and possesses traits observed in other invasive species that are also ecologically generalists (Boudouresque & Verlaque, 2002; Evangelista et al., 2008). During our surveys, we observed that *M*. *macrospora* can grow under a broad range of environmental conditions: (1) in cool to warm waters (~17 to ~30°C); (2) in acidic to basic waters (pH, 5.67–8.34); (3) under shaded (<65 µmol photons m^−2^ s^−1^) and exposed habitats (>500 µmol photons m^−2^ s^−1^); (4) in oligotrophic/mesotrophic waters and highly eutrophic waters (e.g., in Senbaru‐ike Reservoir, Taichung Industrial Park Outlet, and Nanshi river); and (5) on abiotic (e.g., plastic tubes, glasses, or cements) and biotic substrates (e.g., submerged shoots of alive or dead grasses, and snail shells). Our comparison between the climate of the *M*. *macrospora* locations in South America and Taiwan showed that the two environments mainly differ in diurnal and annual temperature oscillations, and precipitation, with overall greater temperature oscillations but less precipitations in Taiwan. This observation further supports that *M*. *macrospora* is an ecologically generalist species. *M*. *macrospora* was reported in Taiwan in the Taoyuan stream from 2005 to 2013 and in the Taichung Industrial Park Outlet from 2013 to 2021, suggesting that its population can self‐sustain and even bloom in new environments for a long period of time.

Our monthly temporal surveys in the Taoyuan stream indicate that constant illumination and strong seasonality (e.g., shorter day length or lower temperature in winter) favor the growth (i.e., percent cover) of *M*. *macrospora*. It is unknown which specific factor plays a more important role in regulating the growth of *M*. *macrospora*, as these factors are highly correlated with each other as confounding factors (e.g., illumination versus day length, and day length versus water temperature). It will be crucial to further examine the relative contribution of these factors on the growth of *M*. *macrospora* in future controlled experiments. Nevertheless, these observations suggest that the areas in East Asia with such environmental properties may have a higher risk of invasion by *M*. *macrospora* from the global aquarium trade. For instance, the open and less‐shaded aquatic ecosystems in higher latitudes (such as Japan, Korea, and northern China) that have strong seasonal patterns are likely most vulnerable to the invasion by *M*. *macrospora*.

Identification of invasive species is important to conservation planning and management, especially in islands. Islands are key hubs of the global aquarium trade (Padilla & Williams, 2004); thus, they are highly vulnerable to the impacts of invasive species due to a high degree of unique biota (Bellard et al., 2017). Having observed that (1) *M*. *macrospora* was the dominant species in the periphyton community in the Taoyuan stream for over a year and that (2) it was extensively growing on other substrates in the Taichung Industrial Park Outlet, we speculate that *M*. *macrospora*, owing to its ecophysiological flexibility, potentially exerts an ecological impact by outcompeting native macroalgae. Recognizing *M*. *macrospora* as an invasive species is the very first step to understand its impact on the invaded regions, such as Taiwan and probably other regions of East Asia.

New species of invasive freshwater macroalgae are rarely reported (Hawes et al., 1991; Kwei & John, 1977; Larkin et al., 2018), unlike species of marine invasive macroalgae that receive a lot more attention (Blanco et al., 2021; Inderjit et al., 2006; Nelson et al., 2021). The lack of studies focusing on invasive freshwater algae leaves a knowledge gap that may hinder conservation planning and management. The global data analyzed in Zhan et al. (2021) revealed that several species of aquarium‐associated freshwater red algae, including *M*. *macrospora*, had the characteristics of an invasive species (i.e., they exhibit an intercontinental distribution and extremely low genetic diversity), suggesting that there may be more species of invasive freshwater macroalgae introduced via the global aquarium trade that have gone undetected and unrecognized. We encourage researchers who specialize in conservation biology and management with an interest in algae ecology to collaborate with phycologists in the future to improve our understanding of the impacts that freshwater algae make on introduced habitats.

## CONFLICT OF INTEREST

The authors have no conflict of interest to declare.

## AUTHOR CONTRIBUTIONS

**Silvia Fontana:** Conceptualization (equal); Data curation (equal); Formal analysis (equal); Methodology (equal); Software (equal); Visualization (equal); Writing – original draft (equal); Writing – review & editing (equal). **Lanwai Yeh:** Data curation (equal); Formal analysis (equal); Methodology (equal); Software (equal); Visualization (equal); Writing – original draft (equal); Writing – review & editing (equal). **Shing Hei Zhan:** Conceptualization (equal); Validation (equal); Visualization (equal); Writing – review & editing (equal). **Shao‐Lun Liu:** Conceptualization (equal); Data curation (equal); Formal analysis (equal); Funding acquisition (equal); Investigation (equal); Methodology (equal); Supervision (equal); Validation (equal); Visualization (equal); Writing – review & editing (equal).

### OPEN RESEARCH BADGES

This article has been awarded Open Data, Open Materials, Preregistered Research Designs Badges. All materials and data are publicly accessible via the Open Science Framework at https://datadryad.org/stash/share/fYc_wy2AVGrDDnfesD8XCX0itVgY6ZDoNIIbDRx_Ig.

## Supporting information

Supplementary Material

## Data Availability

The *cox2*‐*3* sequence newly generated in this study is available at NCBI GenBank under accession numbers: OK442460.
